# Relationship between telomere shortening and age in Korean individuals with mild cognitive impairment and Alzheimer’s disease compared to that in healthy controls

**DOI:** 10.18632/aging.202206

**Published:** 2020-12-15

**Authors:** Eun-Hye Lee, Myung-Hoon Han, Jungsoon Ha, Hyun-Hee Park, Seong-Ho Koh, Seong Hye Choi, Jae-Hong Lee

**Affiliations:** 1 Department of Neurology, Hanyang University Guri Hospital, Guri 11923, South Korea; 2 Department of Neurosurgery, Hanyang University Guri Hospital, Guri 11923, South Korea; 3 GemVax & Kael Co., Ltd, Seongnam 13461, South Korea; 4 Department of Translational Medicine, Hanyang University Graduate School of Biomedical Science and Engineering, Seoul 04763, South Korea; 5 Department of Neurology, Inha University School of Medicine, Incheon 22332, South Korea; 6 Department of Neurology, University of Ulsan College of Medicine, Asan Medical Center, Seoul 05505, South Korea

**Keywords:** aging, telomere length, mild cognitive impairment, Alzheimer’s disease, positron emission tomography

## Abstract

Although telomere length (TL) is highly variable, a shorter TL indicate increased biological age. This multicenter study was conducted to identify the overall correlation between age and TL in Koreans and investigate the associations between age and TL in healthy individuals and patients with mild cognitive impairment (MCI) and Alzheimer’s disease (AD). TL was measured in peripheral leukocyte DNA. MCI and AD were diagnosed based on clinical examinations and amyloid deposition on positron emission tomography. This study enrolled 437 individuals. Multivariable linear analysis showed an overall approximate TL decrease of 37 bp per 1-year increase in age in all individuals (B=-0.037; P=0.002). There was no significant difference in the mean TL between healthy individuals and individuals with AD. Multivariable linear regression analysis showed that the mean rate of telomere shortening was 60 bp per year in individuals with AD (B=-0.060; P=0.006). There was a negative association between age and TL in our study. Our study results showed more significant telomere shortening per year in women than that in men. In addition, individuals with AD had greater telomere shortening every year than healthy individuals and individuals with MCI.

## INTRODUCTION

Telomeres are tandem repeats of the base pairs TTAGGG at the ends of mammalian chromosomes. Telomere length (TL) is an indicator of replicative history and the replicative potential of cells. Therefore, telomeres serve as mitotic clocks [1]. TL is highly variable and the mean TL is a biomarker of aging, with a shorter TL indicating increased biological age [2, 3]. Previous studies have demonstrated varying degrees of negative correlations between age and TL [1, 3–7]. However, to our knowledge, no study has investigated the association between age and TL in Korean individuals. Therefore, we conducted this multicenter study to identify the overall correlation between age and TL in Koreans.

The prevalence of Alzheimer’s disease (AD), a common and severe neurodegenerative disorder, is rapidly increasing in given the aging population worldwide [8]. Several studies have reported an association between a short TL and AD [9–12]. Oxidative stress, inflammation, immune reactions, stress, and microglial cell degeneration in AD have been suggested as risk factors for telomere shortening [13]. In contrast, some studies have reported that TL is not always shorter in patients with AD than in individuals without AD [13–15]. Both short and long TLs have been associated with an increased risk of dementia [15–17]. These inconsistent results make it more difficult to understand the underlying relationship between TL and AD. Therefore, this study aimed compare the association between age and TL in healthy Korean individuals and with that in individuals with mild cognitive impairment (MCI) and AD.

## RESULTS

### Participant characteristics

In total, 437 individuals were enrolled. The mean patient age was 69.9 years, and 58.6% of the participants were female (Table 1). The average TL was longer in female participants than in male participants (8.40 vs. 7.85 kbp; P=0.015). Seventy-five (17.2%) individuals were diagnosed with AD, with female participants showing a higher rate of AD than male participants (21.1% vs. 11.6%, respectively; P=0.003). No significant differences were observed in the prevalence of hypertension, diabetes, hyperlipidemia, heart disease, and stroke between male and female participants. Further detailed information is provided in Table 1.

**Table 1:** Patient characteristics according to sex.

**Characteristics**	**Male**	**Female**	**Total**	**p**
Number (%)	181 (41.4)	256 (58.6)	437 (100.0)	
Age, mean ± SD, years	70.1 ± 8.6	69.9 ± 9.1	69.9 ± 8.9	0.802
Telomere length, mean ± SD, kbp	7.85 ± 2.06	8.40 ± 2.51	8.17 ± 2.35	0.015*
Telomere length, median (IQR), kbp	7.22 (6.54–8.24)	7.53 (6.82–9.11)	7.36 (6.70–8.60)	0.015*
Diagnosis, n (%)				0.003*
N/A	27 (14.9)	39 (15.2)	66 (15.1)	
Normal	67 (37.0)	106 (41.4)	173 (39.6)	
MCI	66 (36.5)	57 (22.3)	123 (28.1)	
AD	21 (11.6)	54 (21.1)	75 (17.2)	
Amyloid-beta PET, n (%)				0.069
N/A	36 (19.9)	76 (29.7)	112 (25.6)	
Negative	91 (50.3)	113 (44.1)	204 (46.7)	
Positive	54 (29.8)	67 (26.2)	121 (27.7)	
Hypertension, n (%)				0.676
No	90 (49.7)	130 (50.8)	220 (50.3)	
Yes	71 (39.2)	93 (36.3)	164 (37.5)	
Missing	20 (11.0)	33 (12.9)	53 (12.1)	
Diabetes, n (%)				0.129
No	128 (70.7)	192 (75.0)	320 (73.2)	
Yes	33 (18.2)	32 (12.5)	65 (14.9)	
Missing	20 (11.0)	32 (12.5)	52 (11.9)	
Hyperlipidemia, n (%)				0.427
No	110 (60.8)	161 (62.9)	271 (62.0)	
Yes	50 (27.6)	61 (23.8)	111 (25.4)	
Missing	21 (11.6)	34 (13.3)	55 (12.6)	
Heart disease, n (%)				0.499
No	142 (78.5)	203 (79.3)	345 (78.9)	
Yes	19 (10.5)	21 (8.2)	40 (9.2)	
Missing	20 (11.0)	32 (12.5)	52 (11.9)	
Stroke, n (%)				0.452
No	152 (84.0)	216 (84.4)	368 (84.2)	
Yes	9 (5.0)	8 (3.1)	17 (3.9)	
Missing	20 (11.0)	32 (12.5)	52 (11.9)	

SD: standard deviation; kbp: kilobase pairs; IQR: interquartile range; N/A: not available; MCI: mild cognitive impairment; AD: Alzheimer’s disease; PET: positron emission tomography. *P<0.05.

### Overall association between age and telomere length

**Figure 1:**
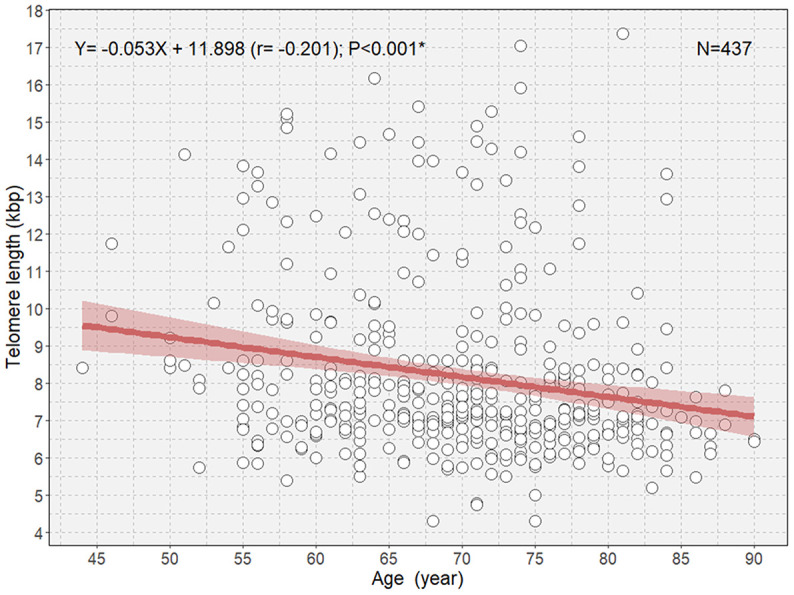
Scatterplot with a linear regression line and 95% confidence interval showing the association between age and telomere length in all participants. *P<0.05.

There was an overall significant negative correlation between age and TL (Figure 1). Multivariable linear regression analysis showed an approximate decrease of 37 bp of TL per 1-year increase in age in participants (B=-0.037; P=0.002; Table 2).

**Table 2:** Multivariable linear regression analysis of telomere length classified by sex according to participant characteristics.

**Variable**	**Multivariable linear regression analysis**					
	**All (n=437)**		**Male (n=181)**		**Female (n=256)**	
	**B (95% CI)**	**P**	**B (95% CI)**	**P**	**B (95% CI)**	**P**
Intercept	10.655		8.889		11.857	
Age	-0.037 (-0.062 to -0.013)	0.002*	-0.016 (-0.051 to 0.019)	0.368	-0.052 (-0.085 to -0.019)	0.002*
MCI	-0.366 (-0.825 to 0.093)	0.118	0.019 (-0.584 to 0.622)	0.951	-0.691 (-1.377 to -0.005)	0.048
AD	-0.138 (-0.687 to 0.411)	0.622	-0.383 (-1.285 to 0.519)	0.403	-0.161 (-0.878 to 0.555)	0.658
Hypertension	-0.214 (-0.636 to 0.207)	0.318	-0.479 (-1.065 to 0.107)	0.108	-0.019 (-0.613 to 0.574)	0.949
Diabetes	-0.027 (-0.570 to 0.517)	0.923	0.146 (-0.567 to 0.859)	0.686	-0.132 (-0.951 to 0.686)	0.750
Hyperlipidemia	-0.144 (-0.587 to 0.300)	0.524	-0.059 (-0.689 to 0.572)	0.854	-0.294 (-0.921 to 0.332)	0.356
Heart disease	0.609 (-0.039 to 1.257)	0.065	0.352 (-0.512 to 1.216)	0.423	0.913 (-0.046 to 1.871)	0.062
Stroke	-0.620 (-1.577 to 0.336)	0.203	-0.696 (-1.890 to 0.498)	0.251	-0.398 (-1.903 to 1.107)	0.602

CI: confidence interval; MCI: mild cognitive impairment; AD: Alzheimer's disease *P<0.05.

### Associations between age and telomere length according to sex

**Figure 2:**
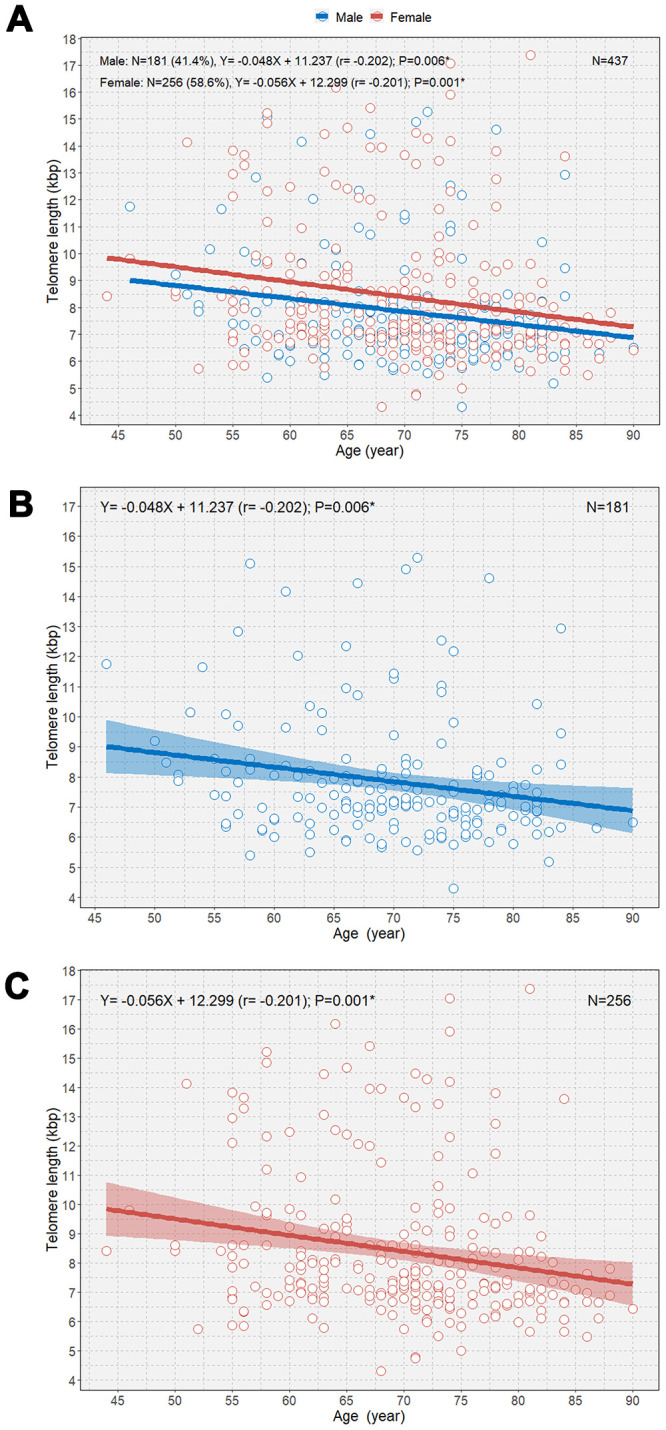
****Scatterplot with linear regression lines with 95% confidence intervals showing the associations between age and telomere length according to sex (**A**), age and telomere length in men (**B**); and age and telomere length in women (**C**). *P<0.05.

**Figure 3:**
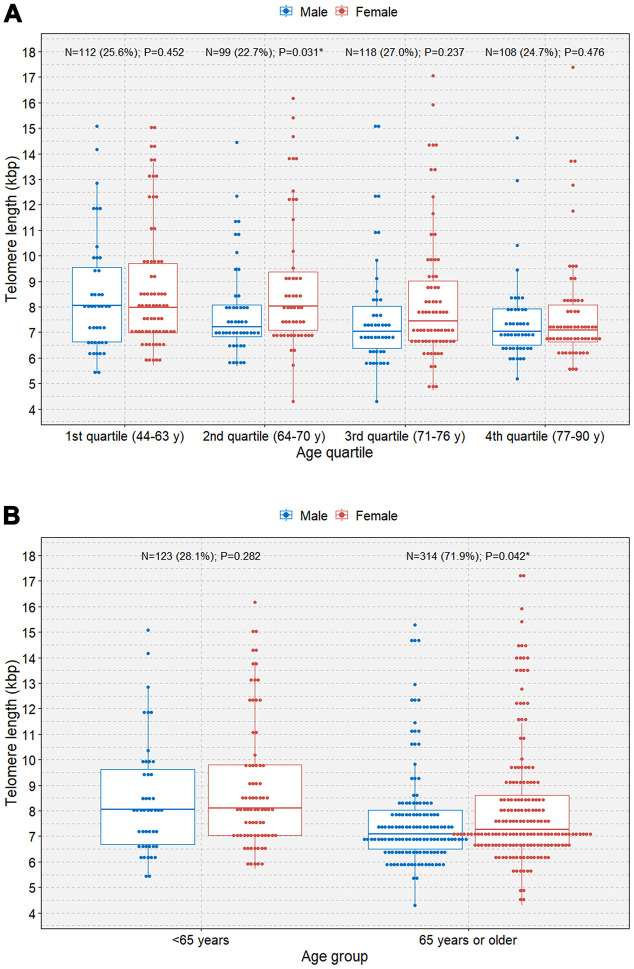
**Boxplots with dot plots of telomere length.** (**A**) telomere length classified by quartile age group according to sex; (**B**) telomere length classified by the 65-year age group according to sex. *P<0.05.

When the study participants were divided according to sex, we observed negative correlations between age and TL in both male and female participants (B=-0.048 [r=-0.202] and P=0.006; B=-0.056 [r=-0.201]; P=0.001, respectively; Figure 2). We observed that female participants had an overall longer TL than male participants and that the rate of telomere shortening was slightly faster in female participants than in participants. However, only female participants showed a significant negative correlation between age and TL in multivariable linear regression analysis (B=-0.052; P=0.002; Table 2). The boxplot shows a statistically significant longer TL in women than in men in the second age quartile (64–70 years; P=0.031; Figure 3A). In the older age group (age ≥65 years), female participants also showed a significantly longer TL than male participants among those (P=0.042; Figure 3B).

### Association between age and telomere length in healthy individuals and in individuals with MCI and AD

**Figure 4:**
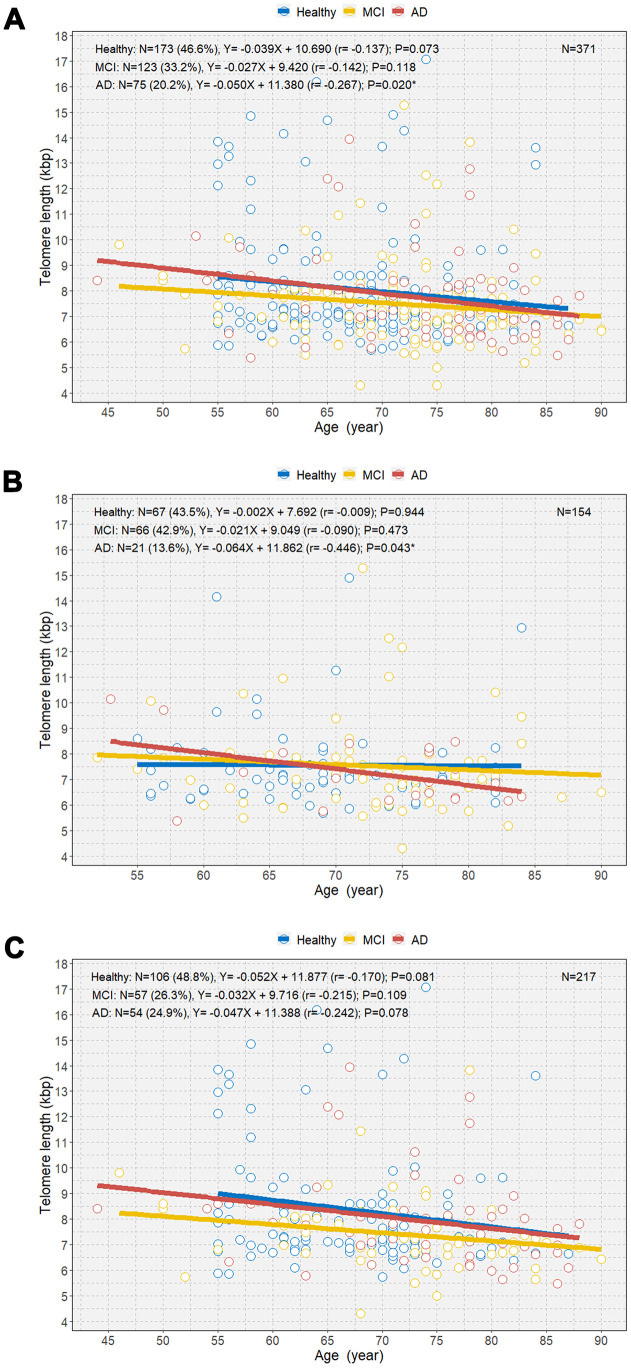
****Scatterplot with linear regression line showing the associations between age and telomere length among healthy individuals and individuals with MCI and AD (**A**), age and telomere length among the three groups in men (**B**), and age and telomere length among the three groups in women (**C**). MCI=mild cognitive impairment; AD=Alzheimer’s disease. *P<0.05.

We observed a negative relationship between age and TL among individuals with MCI and AD and healthy individuals (Figure 4). Overall, individuals with AD demonstrated a significantly steeper negative correlation between age and TL than those with MCI and healthy individuals (B=-0.050 [r=-0.267]; P=0.020; Figure 4A). However, there was no overall significant difference in the mean TL between individuals with AD and healthy individuals (Supplemental Figure 1). Among male participants, only those with AD showed a significant negative correlation between age and TL (B=-0.064 [r=-0.446]; P=0.043; Figure 4B). However, we did not observe significant associations between age and TL in healthy individuals and individuals with MCI and AD (Figure 4C). Multivariable linear regression analysis showed that the mean rate of telomere shortening was 60 bp per year in individuals with AD (B=-0.060; P=0.006) (Table 3). We also observed a linear association between age and TL according to amyloid-beta positron emission tomography (PET) (Supplemental Figure 2). PET-negative and PET-positive individuals showed similar negative associations between age and TL.

**Table 3:** Multivariable linear regression analysis of telomere lengths among healthy individuals and individuals with MCI and AD according to participant characteristics.

**Variable**	**Multivariable linear regression analysis**					
	**Healthy (n=173)**		**MCI (n=123)**		**AD (n=75)**	
	**B (95% CI)**	**P**	**B (95% CI)**	**P**	**B (95% CI)**	**P**
Intercept	10.872		8.999		11.835	
Age	-0.042 (-0.087 to 0.002)	0.063	-0.015 (-0.051 to 0.021)	0.414	-0.060 (-0.102 to -0.018)	0.006*
Hypertension	0.033 (-0.695 to 0.760)	0.929	-0.693 (-1.355 to -0.030)	0.041*	0.291 (-0.560 to 1.142)	0.498
Diabetes	0.059 (-1.000 to 1.117)	0.913	0.051 (-0.727 to 0.829)	0.897	-0.757 (-2.231 to 0.718)	0.309
Hyperlipidemia	-0.156 (-0.911 to 0.599)	0.683	-0.233 (-0.912 to 0.445)	0.498	0.567 (-0.423 to 1.558)	0.257
Heart disease	1.178 (-0.016 to 2.372)	0.053	-0.111 (-1.008 to 0.787)	0.807	1.081 (-0.363 to 2.525)	0.140
Stroke	-1.418 (-3.683 to 0.847)	0.218	-0.841 (-2.067 to 0.385)	0.177	-0.139 (-1.842 to 1.564)	0.871

MCI: mild cognitive impairment; AD: Alzheimer's disease; CI: confidence interval. *P<0.05.

## DISCUSSION

The results of this study demonstrated a mean telomere shortening of approximately 37 bp per 1-year increase in age in Korean individuals after adjusting for medical history. In multivariable analysis, only female participants demonstrated a significant negative association between age and TL. When the study participants were divided into healthy individuals, individuals with MCI, and individuals with AD, only those with AD showed a significant telomere shortening of 60 bp per year in the multivariable model.

In previous studies, the reported mean rates of telomere shortening ranged from 22 to 47 bp per year [1, 3–7]. Studies have reported inconsistent results regarding the degree and rate of telomere shortening with age. One strength of our study is its relatively large sample size. Moreover, to our knowledge, this study is the first study using multicenter data to report the overall association between age and TL in Koreans.

When we classified individuals by sex, the statistically significant negative correlation between age and TL disappeared in men after adjusting for medical history. Similarly, a previous study reported no significant correlation between age and TL in men [18]. The authors suspected that older individuals with poor health may have been less willing to participate in medical research due to their poor health [18]. In addition, men are less likely to participate in research compared to women in general [19]. These findings may explain the lack of relationship between age and TL in men. Our study also showed a higher rate of AD in female participants than in male participants (Table 1).

We observed no significant difference in the mean TL between healthy individuals and individuals with AD. However, there was a more significant telomere shortening every year among individuals with AD than in healthy individuals and those with MCI. Although shorter TLs are associated with AD [20], several studies have reported that TL is not always shorter in patients with AD than in healthy individuals [13–15]. Thus, TL might not be the major determinant of AD because then one might expect that individuals would develop AD as soon as telomeres shorten to a certain size [14]. In addition, a recent study found that both shorter and longer TLs were associated with an increased risk of AD [15]. Our findings demonstrated more rapid telomere shortening per year in individuals with AD than in healthy individuals and those with MCI. Similarly, we also recently showed that a short TL was associated with a rapid decline in cognitive function in patients with MCI and rapid conversion from MCI to AD [21]; however, due to the small sample size, additional replication studies are needed to confirm the study findings.

It is unclear how telomere shortening is associated with AD. It is uncertain whether telomere shortening results directly from AD brain pathology or whether telomere shortening leads to AD [9]. Age had the largest influence on TL in our study. Because old age is the most profound risk factor for AD, individuals with AD may have a higher chance of having short telomeres [22]. However, other explanations have been proposed. For example, oxidative stress contributes to telomere shortening [9, 20, 23]. Oxidative stress results in release of calcium from mitochondria, resulting in mitochondrial dysfunction. Elevated reactive oxygen species can lead to DNA damage, thereby exacerbating telomere shortening [13]. Because AD is associated with oxidative stress, telomere shortening can be accompanied by AD [24]. In addition, microglial cell degeneration is associated with a shorter TL in patients with AD [25]. Microglial activation can contribute to the inflammatory microenvironment and ultimately promote the accumulation of amyloid plaques in AD [26, 27]. In addition, inflammation, immune exhaustion, and chronic stress induced by neurodegenerative diseases are also risk factors for telomere shortening in AD [13].

This study has several limitations. First, the number of enrolled individuals with AD was relatively small. Second, we measured TL in peripheral leukocytes and not in the brain. However, a previous study has shown a direct correlation between TL in peripheral leukocytes and that in the cerebellum [14]. Therefore, we believe that TL in peripheral leukocytes adequately reflected that in the brain. Third, we did not have data regarding the participants’ past medical history, which may have introduced bias. Finally, we could not identify causal relationships owing to the study cross-sectional design.

In conclusion, we estimated the mean rate of telomere shortening per year in Koreans. We identified a negative association between age and TL, which was more prominent in women than in men. We also found a greater rate of telomere shortening in individuals with AD than in those with MCI and healthy individuals. Further studies are needed to better understand the association between age and TL in AD.

## MATERIALS AND METHODS

### Participants

A total of 437 individuals were enrolled from 11 hospitals. These hospitals included nine medical centers that comprised the validation cohort of the Korean Brain Aging Study for the Early Diagnosis and Prediction of AD and two independent centers (Asan Medical Center and Hanyang University Guri Hospital). Participants were diagnosed with MCI if they met the clinical criteria established by the National Institute on Aging-Alzheimer’s Association (NIA-AA) and the modified criteria proposed by Petersen—1) Clinical Dementia Rating (CDR) scale score of 0.5; 2) memory complaints compared to the participant’s previous cognitive function by patients, caregivers, or clinicians; 3) independent activities of daily living; 4) an objective cognitive decline of >1.5 standard deviations below the age-, education-, and sex-adjusted normative means for one or more of the four neuropsychological tests (memory, visuospatial, language, or frontal-executive function) included in the Consortium to Establish a Registry for Alzheimer’s Disease; and 5) absence of dementia. Patients were diagnosed with probable AD according to the criteria proposed by the National Institute of Neurological and Communicative Disorders and Stroke-Alzheimer’s Disease and Related Disorders Association or the NIA-AA; Diagnostic and Statistical Manual of Mental Disorders 4^th^ edition; and CDR scale score of 0.5 or 1. The study individuals included 173 healthy controls, 123 individuals with MCI, and 75 individuals with AD. MCI and AD were diagnosed based on clinical examinations, including Mini Mental State Examination (MMSE), CDR scale, and amyloid-beta PET. Individuals who were diagnosed with other types of dementia, such as frontotemporal dementia, were excluded and classified in the not available group to avoid the risk of bias. The study was performed according to the International Conference on Harmonization Good Clinical Practice guidelines and was approved by the institutional review board of each participating center. Before participation, all participants provided written informed consent. Due to significant cognitive declines in some individuals with AD, informed consent was obtained from their legal guardians.

### Clinical assessments

Individuals were determined to have hypertension if they were prescribed antihypertensive medication(s) or had systolic blood pressure >140 mmHg and diastolic blood pressure >90 mmHg. Diabetes mellitus was diagnosed based on the prescription of insulin or oral hypoglycemic medications, high plasma glucose levels (≥126 mg/dL), or high glycated hemoglobin level (≥6.5%) after 8 hours of fasting. Individuals were diagnosed with hyperlipidemia if they were prescribed lipid-lowering medications or had high levels of total cholesterol (≥200 mg/dL), low-density lipoprotein cholesterol (≥130 mg/dL), and triglycerides (≥150 mg/dL) and low levels of high-density lipoprotein cholesterol (<40 mg/dL). Heart disease was defined as a history of coronary artery bypass grafting, percutaneous coronary intervention, or atrial fibrillation or use of heart-related drugs. Stroke was defined as a medical history of cerebral infarction, intracerebral hemorrhage, or subarachnoid hemorrhage.

### Amyloid-beta positron emission tomography

A total of 325 participants underwent amyloid-beta PET at baseline. The standardized uptake value ratio (SUVR) was calculated using ^18^F-flutemetamol PET and ^11^C-PiB PET, which were adjusted using the pons and the cerebellar gray matter as reference regions, respectively. The composite SUVR was calculated by obtaining the region of interest activity of the frontal, temporal, parietal, occipital, anterior cingulate, and posterior cingulate/precuneus cortices and dividing them by the mean intensity of the reference regions. Amyloid-beta PET positivity was determined based on the composite SUVR (≥0.634 for ^18^F-flutemetamol PET and >1.21 for ^11^C-PiB PET).

### Telomere length assay

At baseline, whole blood from individuals was collected and separated into plasma and buffy coat. The leukocyte DNA was extracted from the buffy coat using D-DEX^™^ II b RBC lysis buffer and D-DEX^™^ II b Cell lysis buffer (Intron, MA, USA). The DNA was then hydrated with 300 μL of DNA hydration solution (QIAGEN, Hilden, Germany). TLs were measured using a nonradioactive TeloTAGGG TL Assay (Roche Boehringer-Mannheim, Grenzach-Wyhlen, Germany) according to the manufacturer’s instructions. Briefly, 2–4 μg of DNA was fragmented using a Hinf I/RsaI enzyme mix and separated using agarose gel electrophoresis. The DNA fragments were then transferred to a nylon membrane (Millipore, Bedford, MA, USA) and incubated with digoxigenin (a digoxigenin-labeled probe), which specifically attaches to telomeric repeats. Next, the membranes were incubated with secondary antibodies conjugated with alkaline phosphatase. TLs were visually measured using chemiluminescence and an image analyzer (ImageQuant LAS 4000, GE Healthcare, Little Chalfont, UK). TLs were determined by comparing them to molecular weight standards.

### Statistical analyses

The chi-square test and Student’s *t*-test were used to identify differences between the two groups. Missing values were replaced with “99” and included in the statistical analysis [28]. Box plots with dot plots were used to assess the differences in TLs according to age groups and sex.

Scatterplots with a regression line were constructed to visualize the associations between age and TL. Multivariable linear regression was performed to evaluate the independent association between age and TL according to sex among individuals with MCI and AD and healthy individuals.

P-values <0.05 were considered statistically significant. All statistical analyses were performed using R software version 3.6.3 and IBM SPSS Statistics for Windows, version 24.0 (Armonk, NY).

## Supplementary Material

Supplementary Figures
