# Supplementary material for: Relationship between telomere shortening and age in Korean individuals with mild cognitive impairment and Alzheimer’s disease compared to that in healthy controls
Source: Aging (Albany NY). 2020 Dec 15;13(2):2089–100. doi: 10.18632/aging.202206 (PMC7880372; doi:10.18632/aging.202206)
Supplement: Supplementary Figures [file aging-13-202206-s001.pdf]

SUPPLEMENTARY FIGURES

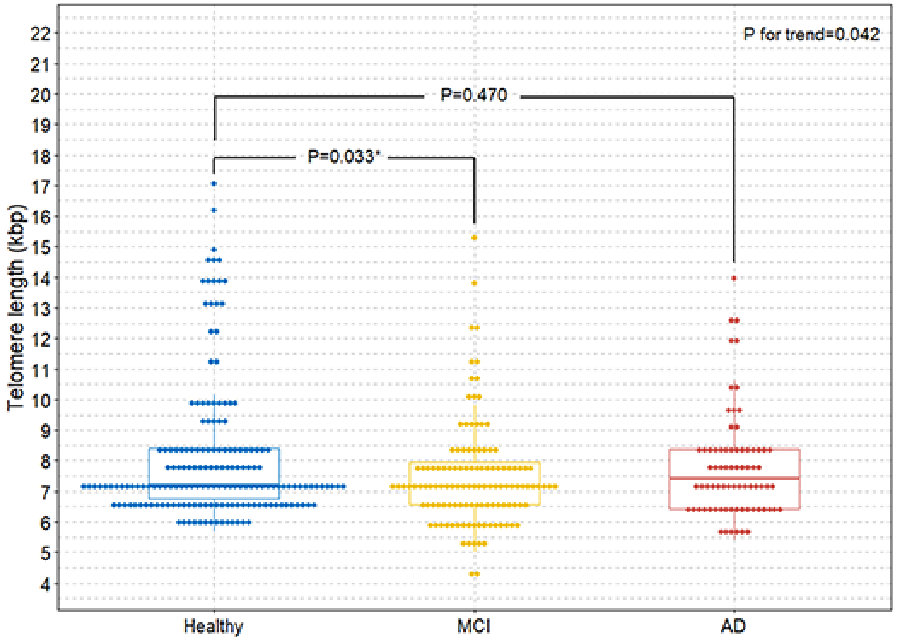

Supplementary Figure 1. Boxplots with dot plots of telomere length among healthy individuals and individuals with MCI and AD. MCI=mild cognitive impairment; AD=Alzheimer's disease *P<0.05.

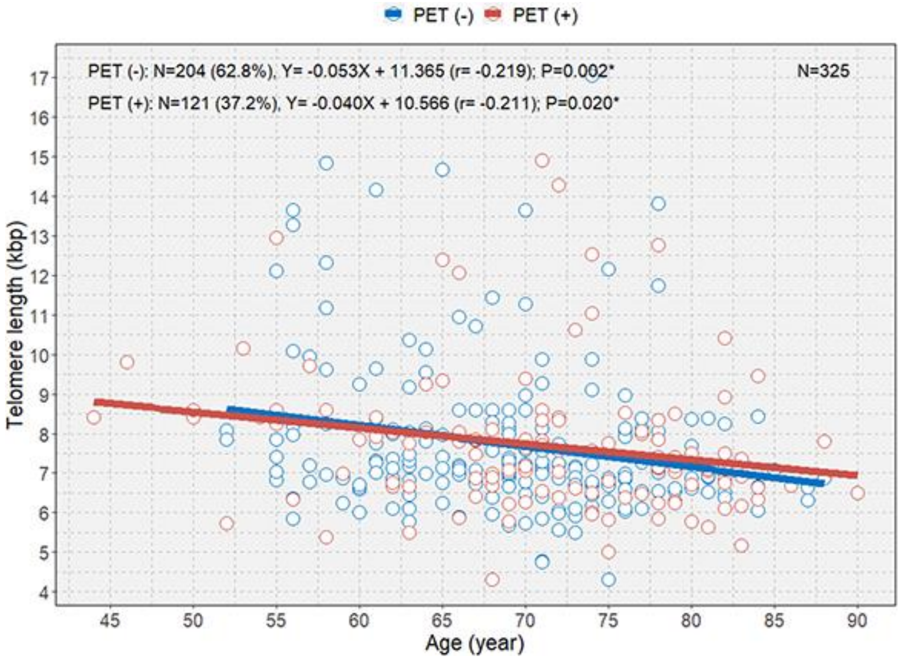

Supplementary Figure 2. Scatterplot with linear regression line showing the association between age and telomere length according to amyloid-beta PET. PET=positron emission tomography *P<0.05.
